# Qualitätsentwicklung im Gesundheitswesen: Sind wir schon so weit, wie wir wollten?

**DOI:** 10.1007/s00103-022-03503-4

**Published:** 2022-03-01

**Authors:** Martin Härter, Uwe Koch-Gromus

**Affiliations:** 1 Zentrum für Psychosoziale Medizin, Institut und Poliklinik für Medizinische Psychologie und Institut für Psychotherapie (IfP), Universitätsklinikum Hamburg-Eppendorf, Martinistraße 52, 20246 Hamburg, Deutschland; 2 Zentrum für Psychosoziale Medizin, Institut und Poliklinik für Medizinische Psychologie, Universitätsklinikum Hamburg-Eppendorf, Martinistraße 5, 20246 Hamburg, Deutschland

Seit Beginn der 1990er-Jahre haben Bemühungen um eine systematische Qualitätssicherung und die Umsetzung von Qualitätsmanagement im deutschen Gesundheitswesen große Bedeutung gewonnen. Initiativen zur Qualitätssicherung beziehen sich dabei auf alle unmittelbar und mittelbar qualitätsrelevanten und -bestimmenden Faktoren im Gesundheitswesen. Neben den strukturellen Voraussetzungen (Strukturqualität) sind hier die Qualität der Ablauforganisation und der Behandlungsprozesse (Prozessqualität) sowie die Güte der tatsächlich erzielten Ergebnisse (Ergebnisqualität) von Bedeutung [1]. Grundprinzip des Qualitätsmanagements ist üblicherweise ein sehr gut bekannter, wiederkehrender bzw. fortlaufender Prozess (sog. Plan Do Check Act oder Deming‑/Shewhart-Zyklus), der gewährleisten soll, dass ein lernendes System entsteht [2, 4].

Differenzierte Qualitätsanforderungen wurden in Deutschland seit Langem sozialrechtlich verankert. So heißt es im Sozialgesetzbuch V: „Sowohl für zugelassene Krankenhäuser als auch für die vertragsärztliche Versorgung bestimmt der Gemeinsame Bundesausschuss (G-BA) sektorenübergreifend durch Richtlinien die verpflichtenden *Maßnahmen der Qualitätssicherung*, die grundsätzlichen *Anforderungen an ein einrichtungsinternes Qualitätsmanagement* sowie *Kriterien für die indikationsbezogene Notwendigkeit und Qualität der durchgeführten diagnostischen und therapeutischen Leistungen*. Dabei sollen auch *Mindestanforderungen an die Struktur-, Prozess- und Ergebnisqualität* festgelegt werden (vgl. SGB V, § 136 Richtlinien des Gemeinsamen Bundesausschusses zur Qualitätssicherung).“

Mit diesen *sektorenübergreifenden Vorgaben zur Qualitätssicherung* löste der G‑BA 2016 seine bisher sektorenspezifisch festgelegten Anforderungen ab. Seitdem gelten für Krankenhäuser, vertragsärztliche, psychotherapeutische und zahnärztliche Praxen weitgehend die gleichen Regeln für die Qualitätssicherung und die Etablierung eines einrichtungsinternen Qualitätsmanagements. Beispielsweise sollen sich Krankenhäuser und Praxen Qualitätsziele setzen und sie regelmäßig kontrollieren. Zudem sollen sie Verantwortlichkeiten festlegen und ein Risiko- und Fehlermanagement durchführen. Die Einrichtungen können bei der Einführung und Umsetzung ihres Qualitätsmanagementsystems eine eigene Ausgestaltung vornehmen oder auf vorhandene Qualitätsmanagementverfahren bzw. -modelle (z. B. DIN-ISO 9001, KTQ, EFQM) zurückgreifen. Auch weitere bedeutsame Einrichtungen im Gesundheitswesen, wie Rehabilitations- und Pflegeeinrichtungen, haben sich um entsprechende Maßnahmen zur Qualitätssicherung zu bemühen.

Eine wachsende Rolle in der Qualitätssicherung spielen darüber hinaus *systematisch entwickelte und evidenzbasierte Leitlinien*, insbesondere der Arbeitsgemeinschaft der Wissenschaftlichen Medizinischen Fachgesellschaften e. V. (AWMF), des Ärztlichen Zentrums für Qualität in der Medizin (ÄZQ), der Deutschen Krebsgesellschaft (DKG) und der Deutschen Krebshilfe, die den fachlichen Wissensstand wiedergeben und bei Behandlungsentscheidungen Ärztinnen und Ärzten, weiteren Therapeutinnen und Therapeuten sowie Patientinnen und Patienten eine Orientierungshilfe bieten. Die Leitlinienarbeit hat einen kulturellen Wandel hin zu systematisch entwickelten Entscheidungshilfen mit Konsensbildung zu relevanten Versorgungsfragen bewirkt. Leitlinien stellen heute eine bedeutsame Wissensgrundlage für zahlreiche Qualitätsinitiativen dar [3].

Nach drei Jahrzehnten etablierter Regelungen zur Qualitätssicherung und allerorten umgesetzter Qualitätsmanagementsysteme im deutschen Gesundheitswesen dürfen zentrale Fragen zur „Wirkung“ dieser Systemeinführung gestellt werden, beispielsweise:

1. Welche Verbesserungen und auch möglichen „Nebenwirkungen“ sind eingetreten?
2. Wie ist das Verhältnis zwischen dem Aufwand und dem Nutzen qualitätssichernder Maßnahmen?
3. Wo bestehen weiterhin Lücken bzw. Entwicklungsbedarf?

Diese Ausgangsfragen hatten uns als Wissenschaftler, die wir der Qualitätssicherung gegenüber positiv eingestellt sind, motiviert, ausgewiesene Expertinnen und Experten aus diesem Anwendungsfeld im Gesundheitswesen zu bitten, Antworten auf diese Fragen aus ihrer Sicht und vor dem Hintergrund unterschiedlicher Verantwortung in diesem Themenfeld zu geben.

Das vorliegende Themenheft beschäftigt sich in 11 Beiträgen zunächst mit den aktuellen Grundlagen der Qualitätssicherung und des Qualitätsmanagements, aber vor allem deren Realisierung in unterschiedlichen Handlungsfeldern der medizinischen Versorgung sowie mit wünschenswerten Entwicklungsperspektiven. Im einführenden Beitrag beschreiben *Dennis Boywitt, Regina Klakow-Franck *und* Claus-Dieter Heidecke* (Berlin) aus der Perspektive des G‑BA und des Instituts für Qualitätssicherung und Transparenz im Gesundheitswesen (IQTIG) Maßnahmen zur Sicherstellung des den gesetzlichen Krankenkassen vorgegebenen Qualitätsgebots. Basierend auf Abrechnungs- und Dokumentationsdaten sowie Patientenbefragungen wird zwischen Fördermaßnahmen, Auswahlentscheidungen und Anreizen für Leistungserbringer unterschieden. Die Autorinnen und Autoren kommen zu der Einschätzung, dass in Deutschland die Möglichkeiten für eine qualitätsorientierte Arzt- und Krankenhauswahl durch Patientinnen und Patienten noch nicht ausreichend umgesetzt sind. Im zweiten Beitrag diskutieren *Cordula Mühr, Frank Brunsmann *und *Martin Danner* (Berlin) aus Sicht der Bundesarbeitsgemeinschaft für Selbsthilfe e. V. die Bedeutung der Patientenperspektive für die Qualitätssicherung in der Medizin. Vor allem bezüglich der Nutzung des Potenzials von regelhaften bzw. kontinuierlichen Patientenbefragungen für die Qualitätssicherung sehen sie in Deutschland noch einen deutlichen Nachholbedarf.

Im Hauptteil des Themenheftes werden die in unterschiedlichen Handlungsfeldern der medizinischen Versorgung inzwischen etablierten Strategien der Qualitätssicherung und des Qualitätsmanagements behandelt. Dabei fokussieren *Max Geraedts* und *Werner de Cruppé* (Marburg) die Qualitätssicherung im Bereich der akutstationären Versorgung. Unter Bezug auf eine international ausgerichtete Literaturrecherche prüfen sie die Frage, ob und welche Effekte sich für die in Deutschland verpflichtenden Qualitätsberichte (QB) und Beteiligungen an externen Qualitätsvergleichen (eQS) nachweisen lassen. Sie finden meist nur schwache Effekte, die am ehesten Verbesserungen bei der Prozessqualität umfassen. Gleichzeitig konstatieren die Autoren einen deutlichen Entwicklungsrückstand in der diesbezüglichen Evaluationsforschung in Deutschland.

*Hans-Jürgen Bartz* (Hamburg) beschreibt und analysiert die Erfahrungen zum klinischen Risikomanagement (kRM) in deutschen Krankenhäusern. Gesetzlich vorgegebene Mindeststandards bestehen für das Risikomanagement, das Fehlermanagement, die Fehlermeldesysteme, das Beschwerdemanagement und für die Nutzung von Checklisten bei operativen Eingriffen. Die geforderte regelmäßige Überprüfung der Wirksamkeit des kRM ist nach Einschätzung des Autors deutlich optimierungsbedürftig.

*Franziska Diel* und *Maurice Rochau* (Berlin) stellen aus Perspektive der Kassenärztlichen Bundesvereinigung (KBV) die Ansätze im Rahmen der vertragsärztlichen Versorgung dar. Sie beschreiben ein enges Netz an zum Teil freiwilligen Maßnahmen und Regeln, die dazu beitragen, dass Patientinnen und Patienten deutschlandweit auf einem hohen qualitativen Niveau vertragsärztlich versorgt werden.

*Ulla Walter* (Hannover) setzt sich mit den Qualitätsentwicklungen in der primären Prävention und Gesundheitsförderung auseinander. Diese erhielten mit der Wiedereinführung der Prävention und Gesundheitsförderung in der gesetzlichen Krankenversicherung und der Verankerung von Qualitätssicherungsmaßnahmen im Präventionsgesetz einen deutlichen Schub. So liegen umfassende Verfahren zur Erfassung von Planungs‑, Struktur‑, Prozess- und Ergebnisqualität sowie entsprechende Handlungsempfehlungen, Checklisten und Instrumente vor. Allerdings wird bisher keines dieser Verfahren flächendeckend und kontinuierlich eingesetzt. Hemmnisse sind unzureichende personelle und finanzielle Ressourcen sowie fehlende Kontinuität bei der Umsetzung der Qualitätssicherung in den Einrichtungen.

*Susanne Weinbrenner et al*. (Berlin) beschreiben, wie in den letzten 25 Jahren in der Rehabilitation der Deutschen Rentenversicherung (DRV) komplexe Verfahren und Instrumente, v. a. der externen Qualitätssicherung, kontinuierlich mit wissenschaftlicher Unterstützung entwickelt und verpflichtend und flächendeckend implementiert wurden. Besonders interessant ist die im Beitrag beschriebene Entscheidung der DRV (und deren Umsetzung), die Zuweisung von Rehabilitanden zu den Rehabilitationskliniken mit den Ergebnissen der externen Qualitätssicherung zu verknüpfen.

*Janine Kleymann-Hilmes, Sophia Brünschwitz *und* Michael Müller *(Berlin) setzen sich in ihrem Beitrag mit der besonderen Bedeutung der Qualitätssicherung in medizinischen Laboratorien und mit deren Risiken auseinander. Das Thema einheitlicher Standards, wie der Vergleichbarkeit der Ergebnisse verschiedener Laboratorien, hat im Kontext der SARS-CoV-2-Pandemie gerade in jüngster Zeit erheblich an Aufmerksamkeit gewonnen. Der Beitrag basiert auf einer Literaturrecherche und Veröffentlichungen zum Qualitätsmanagement sowie auf Erfahrungswerten von Vertretern und Vertreterinnen unterschiedlicher Institutionen.

Die drei Beiträge des abschließenden Themenblockes befassen sich mit übergreifenden Fragestellungen der Qualitätssicherung und des Qualitätsmanagements. Im ersten Beitrag analysieren *Georg Marckmann* (München) und *Jan Schildmann* (Halle) die ethischen Dimensionen der Qualitätssicherung und des Qualitätsmanagements. Dabei wird zum einen die Frage diskutiert, welche ethischen Anforderungen als Qualitätsmerkmale in der Gesundheitsversorgung zu berücksichtigen sind. Anschließend werden ethisch relevante Herausforderungen bei der Bestimmung der Qualität im Gesundheitswesen identifiziert und die Vermittlung professioneller Kompetenzen in der medizinischen Ausbildung als möglicher Beitrag zu Qualität und Qualitätssicherung im Gesundheitswesen erörtert.

*Jan Brönneke *und* Jörg Debatin *(Berlin) setzen sich in ihrem Beitrag mit der Frage auseinander, in welchem Verhältnis der Einsatz verschiedener digitaler Lösungen zu den traditionellen Zielen der Qualitätssicherung in der Gesundheitsversorgung steht. Die Autoren schätzen, dass digitale Lösungen durch die Möglichkeit zur umfassenden Erhebung sowie der zeit- und ortsunabhängigen Bereitstellung von Daten grundsätzlich geeignet sind, Qualität zu sichern. Allerdings ist der Nutzen digitaler Lösungen stark vom jeweils konkreten Anwendungsfall abhängig.

Im letzten Beitrag von *Volker Aßfalg et al.* (München) werden für eine gesetzlich vorgegebene Qualitätssicherungsmaßnahme, das strukturierte Entlassmanagement zur Überleitung von Patientinnen und Patienten aus dem stationären in den ambulanten Sektor, insbesondere die Kosten, die Patientenzufriedenheit und die Wiederaufnahmerate am Beispiel einer universitären chirurgischen Klinik analysiert. Das Entlassmanagement wird als eine effektive Maßnahme bewertet; die dem Krankenhaus entstehenden Mehrkosten werden allerdings erst zeitverzögert im Rahmen der Anpassung der Fallpauschalenvergütung erstattet.

Die Bearbeitung der anspruchsvollen Aufgabenstellung des Themenheftes stellte wegen der Vielfalt und Komplexität von Maßnahmen der Qualitätssicherung und des Qualitätsmanagements im deutschen Gesundheitswesen für uns als Heftkoordinatoren eine inhaltliche Herausforderung dar, da wir eine Auswahl vornehmen mussten, die nicht immer leichtfiel. Wir danken allen Autorinnen und Autoren für die zeitgerechte Erstellung ihrer Manuskripte und die sehr interessanten Einblicke in die unterschiedlichen Facetten zum Thema Qualitätssicherung und Qualitätsmanagement.

Nun wünschen wir Ihnen, liebe Leserinnen und Leser, eine anregende Lektüre,

Ihre

Martin Härter und Uwe Koch-Gromus
